# Estimating the Effects of Hypothetical Alcohol Minimum Unit Pricing Policies on Alcohol Use and Deaths: A State Example

**DOI:** 10.15288/jsad.22-00274

**Published:** 2024-01-23

**Authors:** Lauren Bertin, Gregory Leung, Michele K. Bohm, Jennifer LeClercq, Elizabeth L. Skillen, Marissa B. Esser

**Affiliations:** ^a^ Department of Psychology, Emory University, Atlanta, Georgia; ^b^ Division of Population Health, National Center for Chronic Disease Prevention and Health Promotion, Centers for Disease Control and Prevention, Atlanta, Georgia; ^c^ Policy Analysis and Engagement Office, Office of Policy, Performance, and Evaluation, Centers for Disease Control and Prevention, Atlanta, Georgia

## Abstract

**Objective:**

Alcohol minimum unit pricing (MUP) policies establish a floor price beneath which alcohol cannot be sold. The potential effectiveness of MUP policies for reducing alcohol-attributable deaths in the United States has not been quantitatively assessed. Therefore, this study estimated the effects of two hypothetical distilled spirits MUP policies on alcohol sales, consumption, and alcohol-attributable deaths in one state.

**Method:**

The International Model of Alcohol Harms and Policies tool was used to estimate the effects of two hypothetical MUP per standard drink policies (40-cent and 45-cent) pertaining to distilled spirits products at off-premises alcohol outlets in Michigan during 2020. Prevalence estimates on drinking patterns among Michigan adults were calculated by sex and age group. Prices per standard drink and sales of 9,747 spirits products were analyzed using National Alcohol Beverage Control Association data. Analyses accounted for other alcoholic beverage type sales using cross-price elasticities.

**Results:**

Increasing the MUP of the 3.5% of spirits with the lowest prices per standard drink to 40 cents could reduce total alcohol per capita consumption in Michigan by 2.6% and prevent 232 (5.3%) alcohol-attributable deaths annually. A 45-cent MUP would affect 8.0% of the spirits and reduce total alcohol per capita consumption by 3.9%, preventing 354 (8.1%) deaths.

**Conclusions:**

Modestly increasing the prices of the lowest-priced spirits with an MUP policy in a single state could save hundreds of lives annually. This suggests that alcohol MUP policies could be an effective strategy for improving public health in the United States, consistent with the World Health Organization's recommendation.

Excessive alcohol consumption is among the leading contributing causes of premature mortality in the United States (Woolf & Schoomaker, 2019) and responsible for 140,000 deaths annually (Esser et al., 2022b). There have been recent increases in alcohol-related emergency department visit rates (Esser et al., 2022a) and fully alcohol-attributable deaths (Spencer et al., 2022). The World Health Organization (WHO) and the Community Preventive Services Task Force recommend increasing the price of alcohol through taxation or other pricing policies to reduce excessive drinking and related harms (Elder et al., 2010; WHO, 2022).

In the United States, alcohol taxes are the most common alcohol pricing policy; however, alcohol excise taxes are not inflation-adjusted (Blanchette et al., 2020), contributing to the relative prices of alcoholic beverages becoming less expensive in the past 30 years (Naimi et al., 2018). Minimum pricing policies can complement alcohol taxes and establish a floor price beneath which alcohol cannot be sold (Thompson et al., 2017). Minimum pricing policies can be based on minimum unit pricing (MUP) per standard drink of pure alcohol, or broader and instead based on a container size of a specific beverage type (e.g., liter of beer; LeClercq et al., 2021).

Studies from multiple locations (e.g., United Kingdom, Canada) have shown the effectiveness of minimum pricing policies for reducing alcohol purchases and alcohol-related harms. MUP policies in Scotland and Wales were associated with reductions in alcohol purchases, particularly in households that bought the most alcohol (Anderson et al., 2021), and for commercial retail sales (Giles et al., 2021). Three years after MUP implementation in Scotland, there was a 3% net reduction in retail alcohol sales (Giles et al., 2022). Minimum pricing policies have also been associated with reductions in hospitalizations (Maharaj et al., 2023; Sherk et al., 2020a; Zhao & Stockwell, 2017) and deaths (Wyper et al., 2023; Zhao et al., 2013). The Organisation for Economic Cooperation and Development (OECD) assessed the effectiveness of alcohol interventions and found that minimum pricing policies were the second most effective one, behind increasing taxes (OECD, 2021).

A prospective policy analysis found that minimum pricing policies could be a feasible and effective strategy in the United States (LeClercq et al., 2021). Although they are not common in the United States (ChangeLab Solutions, personal correspondence, December 3, 2018), Oregon used regulatory authority to set minimum prices on distilled spirits (League of Oregon Cities, 2021). The potential effectiveness of alcohol MUP policies for preventing deaths in the United States has not been quantitatively assessed. Therefore, this study estimated the effects of two hypothetical distilled spirits MUP policies on alcohol sales, consumption, and alcohol-attributable deaths in one state. Michigan was selected because detailed data on the prices and sales of spirits are documented since the state controls distilled spirits distribution and sales (Michigan Liquor Control Commission, 2022). Also, the state previously signaled an interest in alcohol pricing policies to reduce alcohol-related harms (Michigan Department of Health and Human Services, 2019, 2020).

## Method

The International Model of Alcohol Harms and Policies (InterMAHP) tool (Canadian Institute for Substance Use Research, 2022) was used to model the effects of two hypothetical MUP policy scenarios pertaining to distilled spirits products sold at off-premises alcohol outlets in Michigan on alcohol-attributable deaths. InterMAHP includes more than 40 alcohol-related conditions, both from the shortterm effects of drinking (e.g., injuries) and from chronic effects (e.g., various cancers; Sherk et al., 2020b). Using population-attributable fractions to calculate the number of alcohol-attributable deaths, InterMAHP integrates relative risk curves (e.g., from WHO) and drinking prevalence estimates and then applies the functions to all deaths from alcohol-related causes (Sherk et al., 2020b). Data on the population's drinking patterns (past-year drinking, former drinking, lifetime abstaining, and past 30-day binge drinking), the number of deaths from alcohol-related causes, and the expected change in alcohol use from a policy intervention were uploaded to InterMAHP.

### Data sources

The Behavioral Risk Factor Surveillance System (BRFSS) is a state-based telephone survey of U.S. adults (≥18 years) that collects health data, including alcohol use (Centers for Disease Control and Prevention [CDC], 2022a). Drinking pattern prevalence estimates were calculated from the 7,269 respondents who answered the alcohol questions in the 2020 Michigan BRFSS (response rate: 48.3%). Past 30-day binge drinking was estimated from the Michigan BRFSS. Since data on the other drinking patterns were not directly available from the BRFSS, national estimates on drinking patterns from the National Survey on Drug Use and Health (NSDUH) were applied to the Michigan BRFSS. The 2019 NSDUH was used because of substantial methodological changes during 2020, including results based on the first and fourth quarters only and multimode data collection during the fourth quarter, with unknown effects on the results (Substance Abuse and Mental Health Services Administration, 2021). Data on the prices and sales of distilled spirits in Michigan during 2020 were obtained from the National Alcohol Beverage Control Association (2022). The prices were based on those designated by the Michigan Liquor Control Commission for sales of distilled spirits products, and the state reports the data to National Alcohol Beverage Control Association. Michigan death data were from the 2020 National Vital Statistics System, compiled from Wide-ranging Online Data for Epidemiologic Research (WONDER; CDC, 2022b). Data were analyzed using Stata MP-17.0 (StataCorp LP, College Station, TX) and uploaded into InterMAHP.

### Prevalence of alcohol consumption patterns by subgroups

Weighted drinking prevalence estimates were calculated by sex for three age groups. For the overall population and sex-specific age groups, the past-year drinking prevalence in Michigan was calculated by multiplying the prevalence of past 30-day drinking reported in the Michigan BRFSS by the national ratio of past-year drinking to past 30-day drinking in the NSDUH. The prevalence of nondrinking in the past year was calculated by subtracting the past-year drinking prevalence from 100%. Then, to calculate the prevalence of lifetime abstaining (no alcohol consumption in lifetime) and former drinking (consumed alcohol in lifetime but not in the past 12 months), past-year nondrinking was stratified based on the proportion of nondrinkers in the NSDUH who reported lifetime abstaining versus former drinking. Past 30-day binge drinking (consumption of ≥5 drinks for males or ≥4 drinks for females, during ≥1 occasions) was calculated from the Michigan BRFSS. Because alcohol use in surveys is underreported relative to alcohol sales data (Stockwell et al., 2018), InterMAHP adjusts the drinking pattern prevalences to avoid underestimating the alcohol-related harms and policy effects (Sherk et al., 2017). The U.S.-specific adjustment factor was used so that alcohol use accounted for 73% of alcohol sales (Esser et al., 2022c). The weighted drinking pattern prevalence estimates are also presented by race/ethnicity, annual household income, education, and county metropolitan status (using the Urban–Rural Classification Scheme for Counties [Ingram & Franco, 2014]). Prevalence estimates were suppressed if results were potentially unstable (i.e., relative standard error >30%; CDC, 2021).

### Distilled spirits products

There were 10,866 distilled spirits products available at off-premises alcohol outlets in Michigan in 2020, spanning 14 major spirits categories. Off-premises alcohol outlets include places such as liquor or convenience stores where alcohol is consumed off-site, as opposed to places where alcohol is consumed on-premises (e.g., bars, restaurants). Descriptive information was provided for each spirits product, including the container size, proof, monthly price, monthly units sold, and the major spirits category (e.g., rum, vodka). In total, 1,119 products were excluded from the study because of implausible or missing values, including 400 products with a listed alcohol by volume (ABV) greater than 100%, a single product with an ABV incorrectly listed as less than 5%, 689 additional products with no pricing data, and 29 with overall negative sales, resulting in 9,747 products in this analysis. Taking the off-premises spirits sales in the database relative to Michigan's 2020 total spirits sales (Slater & Alpert, 2022), the off-premises spirits sales accounted for 92.4% of the spirits sales. This proportion of sales at off-premises outlets was similar to that in the first half of 2021 (90.3%), suggesting that it was not unique to 2020.

The number of standard alcoholic drinks in each product was calculated by converting the container size in milliliters (ml) to ounces. Then, the container size in ounces was multiplied by its percent ABV (e.g., a 25-oz container was multiplied by 0.4 if the ABV was 40%) to calculate the ounces of pure alcohol in each container. A standard U.S. alcoholic drink is defined as 0.6 oz of pure alcohol (U.S. Department of Agriculture & U.S. Department of Health and Human Services, 2020), so the ounces of pure alcohol in each container was divided by 0.6 to calculate each product's number of standard drinks. Each product's price was divided by the number of standard drinks, yielding prices per standard drink. Prices per standard drink were used to assess prices for a fixed amount of alcohol. For products available during more than 1 month of 2020, the average price per standard drink was calculated. To provide an overview of distilled spirits available for retail sales and their prices, the products and average prices per standard drink were analyzed by select product characteristics (e.g., ABV) overall and by spirits category. The 6% sales tax on alcohol sold in Michigan is not included in the database prices because sales taxes are applied at the point of purchase.

### Minimum unit pricing policy scenarios and price elasticities

The two hypothetical MUP policy scenarios for this study were selected by first examining the average prices per standard drink between the first and tenth percentiles to understand the distribution of prices among the inexpensive products, since those products are affected by MUP policies. Second, the number of products that would be affected and the number of sales of the potentially affected products were considered to identify hypothetical MUP scenarios. After examining 5-cent increments between 40 and 65 cents MUPs, 40-cent and 45-cent MUPs were selected to apply to distilled spirits products at off-premises alcohol outlets (higher MUPs did not show greater effects).

The estimated effects of the 40-cent and 45-cent MUP policies on alcohol sales were calculated by assessing the number and percentage of products at off-premises outlets that would be affected, the number and percentage of standard drinks of spirits affected, and the percentage of total alcohol affected (including beer, wine, and distilled spirits sold at on-premises or off-premises alcohol outlets). Detailed data on prices at the time of sale and product-specific sales were only available for spirits at off-premises outlets; therefore, sales of spirits from on-premises outlets, beer, and wine were accounted for using beverage-specific data from alcohol sales, shipments, and production (Slater & Alpert, 2022). The percentage of total alcohol affected was calculated by dividing the estimated number of standard drinks of distilled spirits that would be affected under each MUP policy by the number of standard drinks of total alcohol sold. In Michigan in 2020, approximately 43.5% of all alcohol sold was distilled spirits, 39.5% was beer (including malt liquors), and 16.6% was wine (Slater & Alpert, 2022). It was assumed that the alcohol sold was also consumed in 2020.

Alcohol price elasticities were used to estimate how hypothetical spirits price increases would affect alcohol consumption. Studies in PubMed and Google Scholar with alcohol price elasticities were identified using combinations of the following search terms: *elasticity, alcohol, demand, price, cross-price, own-price*, and *estimating*. Studies were evaluated based on inclusion of own-price and cross-price elasticities by beverage type, geographic location, recency of the estimates, and low risk of bias from funding source. Relevant literature from reference lists was also considered. A study was identified that contained U.S.-based own-price elasticities for spirits (-0.446), beer (-0.370), and wine (-0.710), as well as cross-price elasticities (specifically, Morishima elasticities of substitution) to estimate the percentage change in beer and wine consumption for a given percentage change in the price of spirits (beer-spirits: 0.656; wine-spirits: 0.327; Fleissig, 2021). On request, the author provided the data points underlying the published figures pertaining to elasticities for 2019 (A. Fleissig, personal correspondence, January 27, 2022).

### Alcohol-related deaths

InterMAHP was used to estimate the effects of the MUP policy scenarios on alcohol-attributable deaths among people in Michigan age 15 years or older during 2020 by sex, age, and cause of death category. A sub-analysis in InterMAHP assessed hypothetical policy effects on overall alcohol-attributable deaths by level of average daily alcohol consumption (high consumption was defined as >4 drinks/day for males, >2 drinks/day for females from Esser et al., 2022c, versus lower average consumption). The modelling methodologies assume that the policy was theoretically implemented before 2020 and that the deaths prevented pertain to a single year (Sherk et al., 2020a). Total deaths in Michigan with alcohol-related causes as the underlying cause of death were identified by sex and age group from the National Vital Statistics System using *International Statistical Classification of Diseases and Related Health Problem, 10th Edition* (ICD-10; WHO, 2016) codes (Sherk et al., 2017). Confidence intervals around the death estimates are not produced in InterMAHP, and, therefore, were not included in this study.

## Results

### Michigan drinking patterns

In 2020, the weighted prevalence of past-year drinking in Michigan was 72.0% among males and 70.6% among females, decreasing with age (Table 1). Past-year drinking was least common among non-Hispanic Asian adults (47.3%) and most common among Hispanic adults (83.6%). The past-year drinking prevalence increased with annual household income and education levels. Another 14.8% of males and 13.4% of females formerly drank but not in the past year. Binge drinking in the past 30 days was more common among males (22.1%) than among females (13.2%). Binge drinking prevalence was highest among those who had an income of at least $75,000 and among those who completed some college or technical school.

**Table 1.**
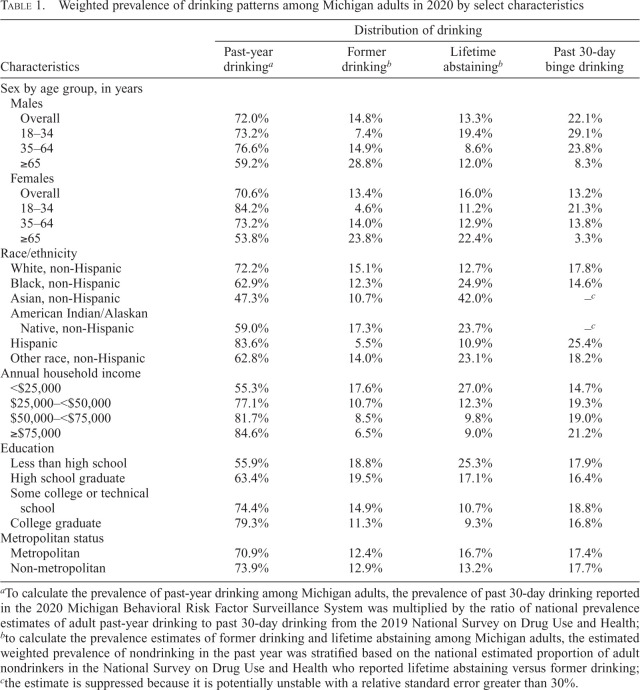
Weighted prevalence of drinking patterns among Michigan adults in 2020 by select characteristics

Characteristics	Distribution of drinking			
	Past-year drinking^a^	Former drinking^b^	Lifetime abstaining^b^	Past 30-day binge drinking
Sex by age group, in years				
Males				
Overall	72.0%	14.8%	13.3%	22.1%
18–34	73.2%	7.4%	19.4%	29.1%
35–64	76.6%	14.9%	8.6%	23.8%
≥65	59.2%	28.8%	12.0%	8.3%
Females				
Overall	70.6%	13.4%	16.0%	13.2%
18–34	84.2%	4.6%	11.2%	21.3%
35–64	73.2%	14.0%	12.9%	13.8%
≥65	53.8%	23.8%	22.4%	3.3%
Race/ethnicity				
White, non-Hispanic	72.2%	15.1%	12.7%	17.8%
Black, non-Hispanic	62.9%	12.3%	24.9%	14.6%
Asian, non-Hispanic	47.3%	10.7%	42.0%	.–^c^
American Indian/Alaskan Native, non-Hispanic	59.0%	17.3%	23.7%	.–^c^
Hispanic	83.6%	5.5%	10.9%	25.4%
Other race, non-Hispanic	62.8%	14.0%	23.1%	18.2%
Annual household income				
<$25,000	55.3%	17.6%	27.0%	14.7%
$25,000–<$50,000	77.1%	10.7%	12.3%	19.3%
$50,000–<$75,000	81.7%	8.5%	9.8%	19.0%
≥$75,000	84.6%	6.5%	9.0%	21.2%
Education				
Less than high school	55.9%	18.8%	25.3%	17.9%
High school graduate	63.4%	19.5%	17.1%	16.4%
Some college or technical school	74.4%	14.9%	10.7%	18.8%
College graduate	79.3%	11.3%	9.3%	16.8%
Metropolitan status				
Metropolitan	70.9%	12.4%	16.7%	17.4%
Non-metropolitan	73.9%	12.9%	13.2%	17.7%

^a^ To calculate the prevalence of past-year drinking among Michigan adults, the prevalence of past 30-day drinking reported in the 2020 Michigan Behavioral Risk Factor Surveillance System was multiplied by the ratio of national prevalence estimates of adult past-year drinking to past 30-day drinking from the 2019 National Survey on Drug Use and Health;

^b^ to calculate the prevalence estimates of former drinking and lifetime abstaining among Michigan adults, the estimated weighted prevalence of nondrinking in the past year was stratified based on the national estimated proportion of adult nondrinkers in the National Survey on Drug Use and Health who reported lifetime abstaining versus former drinking;

^c^ the estimate is suppressed because it is potentially unstable with a relative standard error greater than 30%.

### Distilled spirits products and prices in Michigan

Of the included 9,747 distilled spirits products available at off-premises alcohol outlets in Michigan, the greatest number of products were vodka (2,115), cordials (1,658), and domestic whiskey (1,423) (Table 2). There was a total of 1,970 unique brands. The average price per standard drink was $3.05, and it varied by beverage characteristics. For example, by container size, the average price per standard drink ranged from $1.13 in a 1,000 ml container to $3.95 in a 750 ml container. The average price per standard drink of the 108 products in the lowest cost percentile was $0.33 and the 492 products in the cheapest fifth percentile had an average price per standard drink of $0.44. Median prices per standard drink by ABV and container size are in Supplemental Table A. (Supplemental material appears as an online-only addendum to this article on the journal's website.)

**Table 2.**
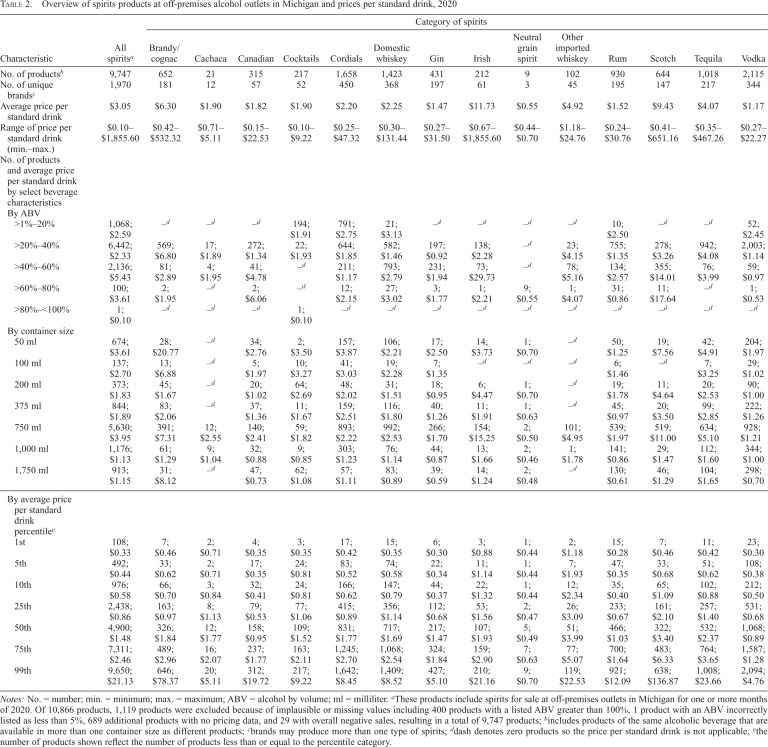
Overview of spirits products at off-premises alcohol outlets in Michigan and prices per standard drink, 2020

Characteristic	Category of spirits														
	All spirits^a^	Brandy/cognac	Cachaca	Canadian	Cocktails	Cordials	Domestic whiskey	Gin	Irish	Neutral grain spirit	Other imported whiskey	Rum	Scotch	Tequila	Vodka
No. of products^b^	9,747	652	21	315	217	1,658	1,423	431	212	9	102	930	644	1,018	2,115
No. of unique brands^c^	1,970	181	12	57	52	450	368	197	61	3	45	195	147	217	344
Average price per standard drink	$3.05	$6.30	$1.90	$1.82	$1.90	$2.20	$2.25	$1.47	$11.73	$0.55	$4.92	$1.52	$9.43	$4.07	$1.17
Range of price per standard drink (min.–max.)	$0.10–$1,855.60	$0.42–$532.32	$0.71–$5.11	$0.15–$22.53	$0.10–$9.22	$0.25–$47.32	$0.30–$131.44	$0.27–$31.50	$0.67–$1,855.60	$0.44–$0.70	$1.18–$24.76	$0.24–$30.76	$0.41–$651.16	$0.35–$467.26	$0.27–$22.27
No. of products and average price per standard drink by select beverage characteristics By ABV															
>1%–20%	1,068; $2.59	–*^d^*	–*^d^*	–*^d^*	194; $1.91	791; $2.75	21; $3.13	–*^d^*	–*^d^*	–*^d^*	–*^d^*	10; $2.50	–*^d^*	–*^d^*	52; $2.45
>20%–40%	6,442; $2.33	569; $6.80	17; $1.89	272; $1.34	22; $1.93	644; $1.85	582; $1.46	197; $0.92	138; $2.28	–*^d^*	23; $4.15	755; $1.35	278; $3.26	942; $4.08	2,003; $1.14
>40%–60%	2,136; $5.43	81; $2.89	4; $1.95	41; $4.78	–*^d^*	211; $1.17	793; $2.79	231; $1.94	73; $29.73	–*^d^*	78; $5.16	134; $2.57	355; $14.01	76; $3.99	59; $0.97
>60%–80%	100; $3.61	2; $1.95	–*^d^*	2; $6.06	–*^d^*	12; $2.15	27; $3.02	3; $1.77	1; $2.21	9; $0.55	1; $4.07	31; $0.86	11; $17.64	–*^d^*	1; $0.53
>80%–<100%	1; $0.10	–*^d^*	–*^d^*	–*^d^*	1; $0.10	–*^d^*	–*^d^*	–*^d^*	–*^d^*	–*^d^*	–*^d^*	–*^d^*	–*^d^*	–*^d^*	–*^d^*
By container size															
50 ml	674; $3.61	28; $20.77	–*^d^*	34; $2.76	2; $3.50	157; $3.87	106; $2.21	17; $2.50	14; $3.73	1; $0.70	–*^d^*	50; $1.25	19; $7.56	42; $4.91	204; $1.97
100 ml	137; $2.70	13; $6.88	–*^d^*	5; $1.97	10; $3.27	41; $3.03	19; $2.28	7; $1.35	–*^d^*	–*^d^*	–*^d^*	6; $1.46	–*^d^*	7; $3.25	29; $1.02
200 ml	373; $1.83	45; $1.67	–*^d^*	20; $1.02	64; $2.69	48; $2.02	31; $1.51	18; $0.95	6; $4.47	1; $0.70	–*^d^*	19; $1.78	11; $4.64	20; $2.53	90; $1.00
375 ml	844; $1.89	83; $2.06	–*^d^*	37; $1.36	11; $1.67	159; $2.51	116; $1.80	40; $1.26	11; $1.91	1; $0.63	–*^d^*	45; $0.97	20; $3.50	99; $2.85	222; $1.26
750 ml	5,630; $3.95	391; $7.31	12; $2.55	140; $2.41	59; $1.82	893; $2.22	992; $2.53	266; $1.70	154; $15.25	2; $0.50	101; $4.95	539; $1.97	519; $11.00	634; $5.10	928; $1.21
1,000 ml	1,176; $1.13	61; $1.29	9; $1.04	32; $0.88	9; $0.85	303; $1.23	76; $1.14	44; $0.87	13; $1.66	2; $0.46	1; $1.78	141; $0.86	29; $1.47	112; $1.60	344; $1.00
1,750 ml	913; $1.15	31; $8.12	–*^d^*	47; $0.73	62; $1.08	57; $1.11	83; $0.89	39; $0.59	14; $1.24	2; $0.48	–*^d^*	130; $0.61	46; $1.29	104; $1.65	298; $0.70
By average price per standard drink percentile^e^															
1st	108; $0.33	7; $0.46	2; $0.71	4; $0.35	3; $0.35	17; $0.42	15; $0.35	6; $0.30	3; $0.88	1; $0.44	2; $1.18	15; $0.28	7; $0.46	11; $0.42	23; $0.30
5th	492; $0.44	33; $0.62	2; $0.71	17; $0.35	24; $0.81	83; $0.52	74; $0.58	22; $0.34	11; $1.14	1; $0.44	7; $1.93	47; $0.35	33; $0.68	51; $0.62	108; $0.38
10th	976; $0.58	66; $0.70	3; $0.84	32; $0.41	24; $0.81	166; $0.62	147; $0.79	44; $0.37	22; $1.32	1; $0.44	12; $2.34	35; $0.40	65; $1.09	102; $0.88	212; $0.50
25th	2,438; $0.86	163; $0.97	8; $1.13	79; $0.53	77; $1.06	415; $0.89	356; $1.14	112; $0.68	53; $1.56	2; $0.47	26; $3.09	233; $0.67	161; $2.10	257; $1.40	531; $0.68
50th	4,900; $1.48	326; $1.84	12; $1.77	158; $0.95	109; $1.52	831; $1.77	717; $1.69	217; $1.47	107; $1.93	5; $0.49	51; $3.99	466; $1.03	322; $3.40	532; $2.37	1,068; $0.89
75th	7,311; $2.46	489; $2.96	16; $2.07	237; $1.77	163; $2.11	1,245; $2.70	1,068; $2.54	324; $1.84	159; $2.90	7; $0.63	77; $5.07	700; $1.64	483; $6.33	764; $3.65	1,587; $1.28
99th	9,650; $21.13	646; $78.37	20; $5.11	312; $19.72	217; $9.22	1,642; $8.45	1,409; $8.52	427; $5.10	210; $21.16	9; $0.70	119; $22.53	921; $12.09	638; $136.87	1,008; $23.66	2,094; $4.76

*Notes*: No. = number; min. = minimum; max. = maximum; ABV = alcohol by volume; ml = milliliter.

^a^ These products include spirits for sale at off-premises outlets in Michigan for one or more months of 2020. Of 10,866 products, 1,119 products were excluded because of implausible or missing values including 400 products with a listed ABV greater than 100%, 1 product with an ABV incorrectly listed as less than 5%, 689 additional products with no pricing data, and 29 with overall negative sales, resulting in a total of 9,747 products;

^b^ includes products of the same alcoholic beverage that are available in more than one container size as different products;

^c^ brands may produce more than one type of spirits;

^d^ dash denotes zero products so the price per standard drink is not applicable;

^e^ the number of products shown reflect the number of products less than or equal to the percentile category.

### Potential effects of minimum unit pricing policies on alcoholic products and consumption

An MUP of 40 cents per standard drink on distilled spirits sold at off-premises alcohol outlets would have increased the prices of 345 distilled spirits products in 2020, about 3.5% of the available spirits products (Table 3). Among those products, the average price per standard drink would need to be increased by less than 6 cents to reach a 40-cent MUP, on average. Those 345 products accounted for 10.2% of the standard drinks of spirits sold (Table 3). By category of spirits, the one with the greatest percentage of total standard drinks potentially affected was vodka (24.8%). Overall, the 40-cent MUP would affect the prices of 4.3% of all standard drinks of alcohol sold when accounting for all types of alcohol (beer, wine, and spirits sold at on-premises or off-premises alcohol outlets).

**Table 3.**
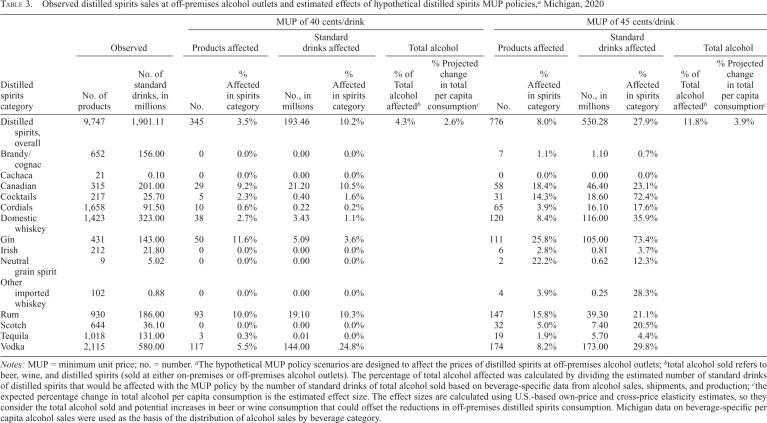
Observed distilled spirits sales at off-premises alcohol outlets and estimated effects of hypothetical distilled spirits MUP policies,^a^ Michigan, 2020

	Observed		MUP of 40 cents/drink								MUP of 45 cents/drink					
			Products affected		Standard drinks affected		Total alcohol		Products affected		Standard drinks affected		Total alcohol	
Distilled spirits category	No. of products	No. of standard drinks, in millions	No.	% Affected in spirits category	No., in millions	% Affected in spirits category	% of Total alcohol affected^b^	% Projected change in total per capita consumption^c^	No.	% Affected in spirits category	No., in millions	% Affected in spirits category	% of Total alcohol affected^b^	% Projected change in total per capita consumption^c^
Distilled spirits, overall	9,747	1,901.11	345	3.5%	193.46	10.2%	4.3%	2.6%	776	8.0%	530.28	27.9%	11.8%	3.9%
Brandy/cognac	652	156.00	0	0.0%	0.00	0.0%			7	1.1%	1.10	0.7%		
Cachaca	21	0.10	0	0.0%	0.00	0.0%			0	0.0%	0.00	0.0%		
Canadian	315	201.00	29	9.2%	21.20	10.5%			58	18.4%	46.40	23.1%		
Cocktails	217	25.70	5	2.3%	0.40	1.6%			31	14.3%	18.60	72.4%		
Cordials	1,658	91.50	10	0.6%	0.22	0.2%			65	3.9%	16.10	17.6%		
Domestic whiskey	1,423	323.00	38	2.7%	3.43	1.1%			120	8.4%	116.00	35.9%		
Gin	431	143.00	50	11.6%	5.09	3.6%			111	25.8%	105.00	73.4%		
Irish	212	21.80	0	0.0%	0.00	0.0%			6	2.8%	0.81	3.7%		
Neutral grain spirit	9	5.02	0	0.0%	0.00	0.0%			2	22.2%	0.62	12.3%		
Other imported whiskey	102	0.88	0	0.0%	0.00	0.0%			4	3.9%	0.25	28.3%		
Rum	930	186.00	93	10.0%	19.10	10.3%			147	15.8%	39.30	21.1%		
Scotch	644	36.10	0	0.0%	0.00	0.0%			32	5.0%	7.40	20.5%		
Tequila	1,018	131.00	3	0.3%	0.01	0.0%			19	1.9%	5.70	4.4%		
Vodka	2,115	580.00	117	5.5%	144.00	24.8%			174	8.2%	173.00	29.8%		

*Notes*: MUP = minimum unit price; no. = number.

^a^ The hypothetical MUP policy scenarios are designed to affect the prices of distilled spirits at off-premises alcohol outlets;

^b^ total alcohol sold refers to beer, wine, and distilled spirits (sold at either on-premises or off-premises alcohol outlets). The percentage of total alcohol affected was calculated by dividing the estimated number of standard drinks of distilled spirits that would be affected with the MUP policy by the number of standard drinks of total alcohol sold based on beverage-specific data from alcohol sales, shipments, and production;

^c^ the expected percentage change in total alcohol per capita consumption is the estimated effect size. The effect sizes are calculated using U.S.-based own-price and cross-price elasticity estimates, so they consider the total alcohol sold and potential increases in beer or wine consumption that could offset the reductions in off-premises distilled spirits consumption. Michigan data on beverage-specific per capita alcohol sales were used as the basis of the distribution of alcohol sales by beverage category.

An MUP of 45 cents per standard drink on distilled spirits sold at off-premises alcohol outlets would have increased the prices of 776 distilled spirits products in 2020, about 8.0% of the available spirits products (Table 3). Among those products, the average price per standard drink would need to be increased by less than 13 cents to reach a 45-cent MUP, on average. Those 776 products accounted for 27.9% of the standard drinks of spirits sold (Table 3). By category of spirits, the two with the greatest percentage of total standard drinks potentially affected include gin (73.4%) and cocktails (72.4%). Overall, the 45-cent MUP would affect the prices of 11.8% of all standard drinks of alcohol sold.

After applying the own-price elasticity estimates for distilled spirits only, the expected percentage change in the consumption of distilled spirits from off-premises outlets is -7.6% for the 40-cent MUP and -17.8% for the 45-cent MUP. However, given potential increases in beer or wine consumption that could offset the reductions in off-premises distilled spirits consumption, beverage-specific own-price and cross-price elasticity estimates were then applied, yielding an expected percentage change in total alcohol per capita consumption of -2.6% with the 40-cent MUP and -3.9% with the 45-cent MUP (Table 3). An apparent threshold effect was observed such that with each 5-cent increase in the MUP policy scenario between a 45-cent MUP and 65-cent MUP, there was a smaller expected percentage reduction in total alcohol per capita consumption (e.g., -3.7% with the 55-cent MUP, -3.0% with the 60-cent MUP, -2.0% with the 65-cent MUP).

### Potential effects of MUP policy scenarios on alcohol-attributable deaths

**Figure 1.**
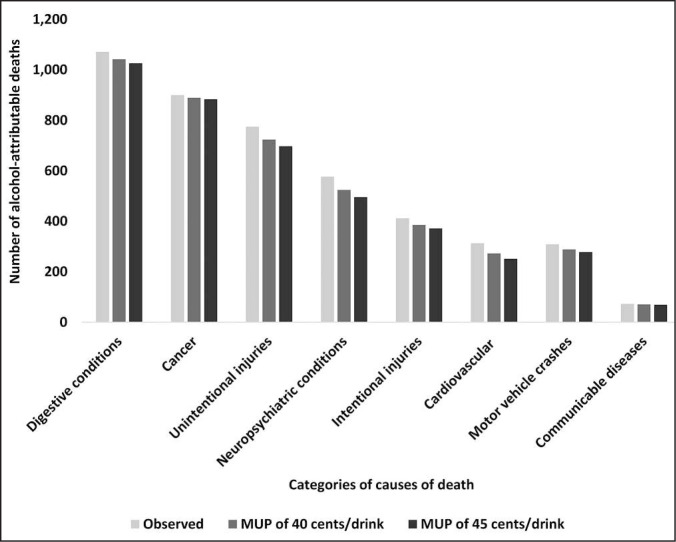
Alcohol-attributable deaths by category of cause of death with harmful effects of alcohol^a^ in Michigan in 2020, observed and estimated by minimum unit price (MUP) policy scenario. ^a^Endocrine conditions were assessed in addition to the categories of causes of death shown, and there were no estimated harmful effects of alcohol use.

There were 4,394 alcohol-attributable deaths in Michigan in 2020 (males: 3,067; females: 1,327), calculated in InterMAHP (Table 4). If a 40-cent MUP policy had been implemented, it could have prevented 232 deaths in 2020 (-5.3%), assuming a 2.6% reduction in total alcohol per capita consumption relative to the observed. A 45-cent MUP could have prevented 354 deaths (-8.1%), assuming a 3.9% reduction in total alcohol per capita consumption. The MUP policies would prevent more deaths among males than among females, with the greatest number of deaths prevented among males ages 35–64 years. Digestive conditions, cancer, and unintentional injuries were the leading causes of alcohol-attributable deaths in Michigan (Figure 1). Across the causes of deaths, a 45-cent MUP could prevent a greater number of deaths than a 40-cent MUP.

**Table 4.**
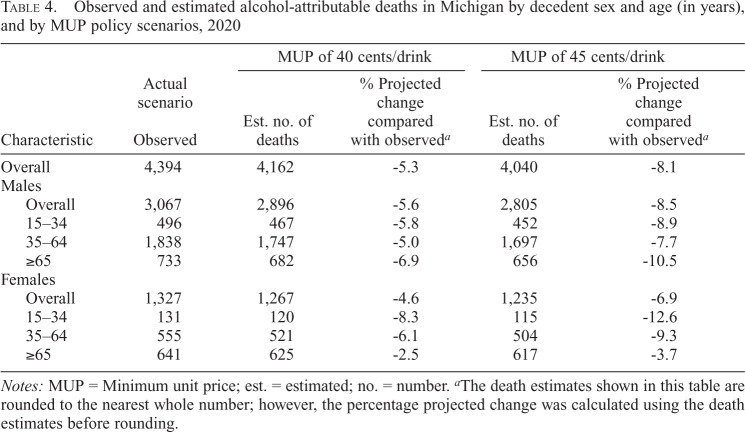
Observed and estimated alcohol-attributable deaths in Michigan by decedent sex and age (in years), and by MUP policy scenarios, 2020

Characteristic	Actual scenario Observed	MUP of 40 cents/drink		MUP of 45 cents/drink	
		Est. no. of deaths	% Projected change compared with observed^a^	Est. no. of deaths	% Projected change compared with observed^a^
Overall	4,394	4,162	-5.3	4,040	-8.1
Males					
Overall	3,067	2,896	-5.6	2,805	-8.5
15–34	496	467	-5.8	452	-8.9
35–64	1,838	1,747	-5.0	1,697	-7.7
≥65	733	682	-6.9	656	-10.5
Females					
Overall	1,327	1,267	-4.6	1,235	-6.9
15–34	131	120	-8.3	115	-12.6
35–64	555	521	-6.1	504	-9.3
≥65	641	625	-2.5	617	-3.7

*Notes:* MUP = Minimum unit price; est. = estimated; no. = number.

^a^ The death estimates shown in this table are rounded to the nearest whole number; however, the percentage projected change was calculated using the death estimates before rounding.

A sub-analysis of deaths by drinking level showed that most of the alcohol-attributable deaths that could be prevented would be among people who drink at high average daily levels (118 of the 232 preventable deaths [50.9%] for the 40-cent MUP scenario; approximately 180 of the 354 preventable deaths [51.0%, based on unrounded death counts] for the 45-cent MUP scenario).

## Discussion

The current study found that increasing the price of the distilled spirits products in Michigan with the lowest prices per standard drink by an average of 6 cents or 13 cents to reach the 40-cent and 45-cent MUPs, respectively, could reduce alcohol-attributable deaths by about 5% or 8% in 1 year, depending on the MUP. These MUPs work by increasing the total shelf price of alcoholic beverages. For example, a common distilled spirits product is one with a 40% ABV in a 750 ml container. The one with the lowest price per standard drink (29.5 cents) had a shelf price of $4.99 in 2020; its new shelf price would be $6.76 with the 40-cent MUP or $7.61 with the 45-cent MUP.

Although it is possible for real-world evaluations to differ from modeled projections, a recent multi-country systematic review found that the modeled effects of MUP policies on alcohol-related hospitalizations were generally consistent with the natural experiment evaluation results (Maharaj et al., 2023). Minimum pricing policy effects can also vary over time. For example, during the initial months after MUP was implemented in Scotland, decreases in road traffic crashes were observed (Vandoros & Kawachi, 2022), but this effect was no longer significant after 2 years (Manca et al., 2022).

### Policy implementation considerations

There are several factors related to minimum pricing policy implementation to consider. The minimum pricing policy could pertain to one alcoholic beverage type (e.g., only distilled spirits) or more than one (e.g., distilled spirits, beer, and wine). In this study, MUP policy scenarios above 45 cents were not associated with greater projected reductions in total alcohol consumption or alcohol-related deaths because people could potentially switch from drinking distilled spirits to drinking beer or wine with increasing prices of the lowest-cost spirits. Beverage substitution might be expected if minimum pricing policies do not apply to all beverage types (Thompson et al., 2017). Also, compared with broader minimum pricing policies, MUP policies tend to have a lower risk of incentivizing the consumption of products with high alcohol content yet relatively low costs per standard drink (Thompson et al., 2017).

Minimum pricing policies could only apply to products at off-premises alcohol outlets (e.g., liquor or convenience stores) or also to products at on-premises outlets, based on each state's license types. Alcohol sales at on-premises outlets might be less responsive to changes in price than at off-premises outlets (Sharma et al., 2017).

To inform optimal minimum pricing levels, surveillance of alcohol pricing and sales data that are collected and reported using standardized methods would be valuable to assess the quantity of products sold with the lowest prices per standard drink. An accessible, comprehensive, jurisdiction-specific surveillance system of alcoholic beverage prices for U.S. states does not exist. Prior studies with actual prices of alcoholic products are at least a decade old (Albers et al., 2013; DiLoreto et al., 2012). Much of the alcoholic beverage pricing data collected come from states that control the distribution and sales of one or more specific alcoholic beverage types, but prices likely differ in license states that do not control sales (e.g., distilled spirits prices might be lower; Siegel et al., 2013). Expanded use of retail scanner data might be helpful for surveillance of alcohol pricing and product-specific sales.

Jurisdiction-specific circumstances might also be considered such as the prices of products from local producers versus imported products and other existing alcohol policies, such as those summarized in the Alcohol Policy Information System (National Institute on Alcohol Abuse and Alcoholism, 2022). For example, policy loopholes could be assessed, such as policies that allow retailers to sell alcohol below the established prices with volume discounts. Stakeholder engagement might also be part of the implementation process. Some retailers might have initial concerns or confusion about MUP policies, although some concerns might be mitigated through clear policy implementation guidance and discussions about policy implications (Stead et al., 2023).

### Health equity considerations

The effects of MUP policies on alcohol sales, consumption, and harms could differ by individuals’ age, gender, and income, largely driven by differences in drinking patterns. This study showed that MUP policies would prevent more deaths among males than among females, with the largest number of deaths prevented among males ages 35–64 years. It also found that more deaths would be prevented among people who drink alcohol at high average levels compared with those drinking at lower levels. However, MUP might not reduce drinking or alcohol-attributable deaths among the smaller percentage of people who already have an alcohol use disorder (Public Health Scotland, 2022). Although data on the type of alcohol or specific alcoholic products consumed were not available for the U.S. context to assess differences by other characteristics, such as income, other studies have shown that people who have lower income levels and drink at higher levels are more likely to purchase the lower-cost products affected by the MUP policies (Holmes et al., 2014). One study found that people in lower-income households in England would not increase their total expenditures on alcohol after an MUP was implemented but would instead purchase less alcohol (Anderson et al., 2021). In addition, studies have shown that people with lower incomes tend to experience a greater burden of alcohol-related harm compared with people with higher incomes, despite drinking similar or lower amounts of alcohol (Boyd et al., 2022). Therefore, people with lower incomes may be more likely to experience the public health benefits of MUP policies, such as with reductions in alcohol-related hospitalizations and deaths (Holmes et al., 2014; Zhao & Stockwell, 2017). As such, MUP policies might reduce alcohol-related health disparities (Meier et al., 2016).

### Limitations

Several study limitations exist. This study used the prices of distilled spirits products set by the Michigan Liquor Control Commission for off-premises alcohol outlets since the exact prices for which off-premises outlets sold the products to customers are not accessible using this data source. Also, this analysis calculated the proportion of spirits sold at off-premises versus on-premises outlets during 2020. Although the proportion was similar to that in the first half of 2021, it could decline as more time passes since COVID-19 mitigation strategies were in place. If the percentage of spirits consumed off-premises were to decrease slightly but the proportion of total spirits sold (including both on-premises and off-premises) relative to total alcohol sold remains similar, it could have minimal impact on the estimated MUP effects on changes in total per capita alcohol consumption. However, if the proportion of spirits sold relative to other alcoholic beverages changes, estimated MUP effects could differ with future years of data.

The 45-cent MUP level in this study associated with the greatest reductions in total alcohol sales and consumption may not be generalizable to other states or contexts given different population-level drinking patterns, distilled spirits prices, and the year of data. Relatedly, price elasticities change over time (Fleissig, 2021), and pricing policies—including MUP—would ideally be adjusted for inflation (Thompson et al., 2017). Also, this study did not account for other factors that affect the prices consumers paid, such as the sales tax on alcohol or pricing promotions, or use Michigan-specific price elasticity estimates, so the estimated effects of the MUP policies could differ if these factors were assessed. It is possible that some of the distilled spirits sold were not consumed by Michigan residents, but it is also possible that Michigan residents bought alcohol in other states; therefore, this is not likely to meaningfully affect the findings. Further, data were not available on the prices of beer or wine, so it was assumed that the prices of those alcoholic beverages remained constant when estimating the potential shift from spirits consumption to beer and wine. However, with increases in the prices of inexpensive spirits, prices of other products could also change. Last, among both males and females, adults age 65 years and older reported a greater prevalence of lifetime alcohol abstention than adults ages 35–64. Therefore, some people could have been misclassified as having abstained from alcohol during their lifetime but formerly drank, which could contribute to underestimating alcohol-related harm (Callinan et al., 2019).

### Conclusions

This study is an example of how MUP policy effects can be calculated using available data for one state. It demonstrates the potential effectiveness of alcohol MUP policies for reducing alcohol sales, drinking, and deaths. The estimated reduction in deaths is conservative because it only pertains to a single year and does not account for deaths prevented over a longer term. However, given that unique jurisdictional implementation considerations exist, jurisdiction-specific analyses could further inform MUP policy development and implementation. MUP policies provide jurisdictions with a complementary strategy for addressing the affordability of alcohol, in addition to increasing alcohol taxes (WHO, 2022).
